# Structural- and DTI- MRI enable automated prediction of IDH Mutation Status in CNS WHO Grade 2–4 glioma patients: a deep Radiomics Approach

**DOI:** 10.1186/s12880-024-01274-9

**Published:** 2024-05-03

**Authors:** Jialin Yuan, Loizos Siakallis, Hongwei Bran Li, Sebastian Brandner, Jianguo Zhang, Chenming Li, Laura Mancini, Sotirios Bisdas

**Affiliations:** 1 Department of Radiology, Shenzhen People’s Hospital, Second Clinical Medical College of Jinan University, First Affiliated Hospital of Southern University of Science and Technology, Shenzhen, China; 2 Queen Square Institute of Neurology, University College London, London, UK; 3 Department of Informatics, Technical University of Munich, Munich, Germany; 4 Martinos Center for Biomedical Imaging, Massachusetts General Hospital, Harvard Medical School, Charlestown, USA; 5 Division of Neuropathology, Queen Square Institute of Neurology, University College London, London, UK; 6 Department of Computer Science and Engineering, Southern University of Science and Technology, Shenzhen, China; 7 Lysholm Department of Neuroradiology, University College London Hospitals NHS Foundation Trust, London, UK

**Keywords:** IDH, Mutation status, Prediction, DTI, Deep neural network, Supervised learning, CNN, SVM

## Abstract

**Background:**

The role of isocitrate dehydrogenase (IDH) mutation status for glioma stratification and prognosis is established. While structural magnetic resonance image (MRI) is a promising biomarker, it may not be sufficient for non-invasive characterisation of IDH mutation status. We investigated the diagnostic value of combined diffusion tensor imaging (DTI) and structural MRI enhanced by a deep radiomics approach based on convolutional neural networks (CNNs) and support vector machine (SVM), to determine the IDH mutation status in Central Nervous System World Health Organization (CNS WHO) grade 2–4 gliomas.

**Methods:**

This retrospective study analyzed the DTI-derived fractional anisotropy (FA) and mean diffusivity (MD) images and structural images including fluid attenuated inversion recovery (FLAIR), non-enhanced T1-, and T2-weighted images of 206 treatment-naïve gliomas, including 146 IDH mutant and 60 IDH-wildtype ones. The lesions were manually segmented by experienced neuroradiologists and the masks were applied to the FA and MD maps. Deep radiomics features were extracted from each subject by applying a pre-trained CNN and statistical description. An SVM classifier was applied to predict IDH status using imaging features in combination with demographic data.

**Results:**

We comparatively assessed the CNN-SVM classifier performance in predicting IDH mutation status using standalone and combined structural and DTI-based imaging features. Combined imaging features surpassed stand-alone modalities for the prediction of IDH mutation status [area under the curve (AUC) = 0.846; sensitivity = 0.925; and specificity = 0.567]. Importantly, optimal model performance was noted following the addition of demographic data (patients’ age) to structural and DTI imaging features [area under the curve (AUC) = 0.847; sensitivity = 0.911; and specificity = 0.617].

**Conclusions:**

Imaging features derived from DTI-based FA and MD maps combined with structural MRI, have superior diagnostic value to that provided by standalone structural or DTI sequences. In combination with demographic information, this CNN-SVM model offers a further enhanced non-invasive prediction of IDH mutation status in gliomas.

## Introduction

Mutations in the isocitrate dehydrogenase (IDH) genes define the brain tumour types IDH-mutant astrocytoma central nervous system (CNS) World Health Organization (WHO) grade 2, 3, and 4 and oligodendroglioma CNS WHO grade 2 and 3 (previously termed diffuse Astrocytoma IDH-mutant WHO grade II, Anaplastic astrocytoma WHO Grade III, and Glioblastoma IDH-mutant WHO Grade IV and oligodendroglioma or anaplastic oligodendroglioma WHO grade II or III, respectively) [1]. The IDH mutation is linked to the pathogenesis of these two tumour types and is thought to increase the intracellular hypoxia-inducible factor-1α, which induces angiogenesis and mediates the evasion of tumour cells [2–4]. Patients with IDH-mutant low-grade gliomas (LGGs) can have better prognosis compared with those with wildtype IDH status. This is pronounced when the IDH-wildtype status is linked to a telomerase reverse transcriptase (TERT) promoter mutation, chromosome 7 trisomy and chromosome 10 monosomy, and possibly also epidermal growth factor receptor (EGFR) amplification, which are diagnostic features of glioblastoma, IDH-wildtype, CNS WHO grade 4 [5]. Targeted drugs and vaccines focusing on IDH mutation have introduced the era of targeted therapy in gliomas [6, 7]. Therefore, IDH status prediction is essential not only for the diagnosis but also for prognostication and treatment planning of patients with gliomas. Determination of IDH mutation status is usually performed on biopsy material, i.e. invasively, using either a mutation-specific antibody on tissue section, or nucleic acid-based test methods, such as Sanger sequencing or next generation sequencing methods, or (indirectly) deoxyribonucleic acid (DNA) methylation arrays. Although current trends in patient management favour radical surgery, treatment selection depends on individual patient characteristics as well as risk estimation [8–12]. More importantly, surgical outcomes can depend on tumour molecular characteristics and predominantly on IDH mutation status [12, 13]. It is therefore advantageous if IDH mutation status can be characterized in advance of treatment decision making, ideally with efficient non-invasive tools. To this end, image analysis enhanced by artificial intelligence has provided promising outcomes [14, 15].

Considerable attempts have been made to identify non-invasive imaging biomarkers for IDH-mutation status prediction [10, 16, 17]. Initial evidence shows that magnetic resonance spectroscopy (MRS) can quantify the 2-hydroxyglutarate (2-HG) levels and enable the non-invasive identification of IDH mutant gliomas [18], but the rates of false positives and false negatives can be high when the tumour has low-to-moderate cellularity or when necrosis to tumour ratio is high [19–21, 3, 22]. In terms of vascular proliferation, magnetic resonance (MR) perfusion can gauge the degree of neo-angiogenesis and potentially p**redi**ct IDH mutation status [19, 23–25]. However, the proposed methodology yielded moderate discriminative power, likely due to the highly variable perfusion patterns in gliomas and the high variability of acquisition and analysis protocols across several participating centres [26].

Diffusion tensor imaging (DTI) is an advanced imaging technique that allows white matter fibre tracking for preoperative planning as well as for tumour grading and differentiation of tumour recurrence from radiation necrosis [27]. A previous study showed that preoperative B0 and fractional anisotropy (FA) have potential value in discriminating IDH-mutant from IDH-wildtype WHO grade 2 or 3 gliomas [10]. Similarly, Xiong et al. reported significantly higher minimal apparent diffusion coefficient (ADC) values and lower maximal FA values in oligodendroglial IDH-mutant tumours compared with their IDH wildtype counterparts [28]. However, those DTI studies only addressed the diagnostic task in certain grades or types of gliomas, rather than the whole range of histologically proven CNS WHO grade 2–4 gliomas. Limited radiogenomics research studies investigate the role of DTI, compared with structural magnetic resonance imaging (MRI), for the non-invasive classification of IDH status based on the profiles of invasiveness, higher angiogenesis, and cell proliferation [29–31]. Meanwhile, some studies evaluating the value of diffusion kurtosis imaging (DKI) suggested DKI was superior to DTI in IDH genotype prediction [32–34]. However, the numbers of cases studied were comparatively small, and the applied DKI algorithm was based on isotropic diffusion acquisitions which may potentially introduce inaccuracy into the kurtosis tensor estimation.

In the current study, we sought to solidify current evidence on the efficacy of DTI to non-invasively detect IDH-mutation status across various glioma grades (CNS WHO grades 2–4). To this end, we built upon recent advances in AI and combined a support vector machine (SVM) classifier with feature differentiable, replacing complex human-designed pipelines. The resulting convolutional neural networks (CNN) -SVM differentiable model incorporated DTI and structural images of different from multiple scanners and allowed both comparative and combinatory assessment of datasets. With this model, we aspire to provide a versatile tool, applicable for non-invasive IDH-mutation status prediction in diverse settings.

## Materials and methods

### Patient population

This retrospective study was approved by the local Research Ethics Committee (University College London / University College London Hospitals Joint Research Office: Reference number 213920, North West - Liverpool Central Research Ethics Committee: Reference number: 18/NW/0395) and written informed consent was waived. Institutional and departmental databases were queried between February 2009 to February 2021 from the Department of Neuroradiology, University College London Hospitals NHS Foundation Trust, London to find all adult patients (age ≥ 18 years) with histologically confirmed CNS WHO grade 2–4 gliomas. Patients’ data were included based on the availability of (a) histopathologically confirmed IDH mutational status, (b) pre-treatment MRI including routine structural sequences and DTI. We excluded cases with unavailable structural imaging, corrupt images or motion artefacts, and inconclusive histological and molecular diagnosis.

### IDH mutation diagnosis

Histomolecular characterization of the gliomas included in this retrospective study was based on the 2016 WHO classification of CNS tumour and the cIMPACT-Now guidelines, at the Department of Neuropathology, University College London Hospitals NHS Foundation Trust, London, including the tumour molecular profiles of IDH mutation status, 1q/19q codeletion status, O^6^-methylguanine-DNA methyltransferase (MGMT) promoter methylation status, EGFR amplification, TERT promoter, alpha-thalassemia/mental retardation X-linked (ATRX) mutation status [35, 36]. IDH1/2 and TERT promoter mutational status were confirmed by Sanger sequencing [37, 38]. Molecular markers including 1q/19p codeletion status, MGMT, EGFR amplification were tested using a quantitative polymerase chain reaction (qPCR) assay [19].

### Image acquisition

All images were acquired on three clinical 3.0 Tesla MR scanners (Trio Tim, Skyra and Prisma, Siemens Healthineers, Erlangen, Germany) with 32- or 64-channel head coils. Specifically, there were 112 cases from the Trio Tim scanner, 12 cases from the Skyra, and 82 cases from the Prisma scanner. The acquisition parameters of the three-dimensional (3D)T1-, T2- weighted images and fluid attenuation inversion recovery (FLAIR) images were as follows: repetition time (TR) = 510–600 ms, echo time (TE) = 30–37 ms for T1-weighted images (T1WI); TR = 4100–4780 ms, TE = 86–98 ms for T2-weighted images (T2WI); TR = 1000 ms, TE = 45 ms, inversion time = 800–810 ms for FLAIR images. The field of view (FOV) was 200 × 200 mm^2^ and a matrix of 200 × 200. The DTI acquisition parameters were as follows: a single-shot (SS) spin-echo (SE) echo-planar imaging (EPI) sequence was used with TR = 7600-9400ms, TE = 70–84 ms, b-values = 1000–3000 s/mm^2^. The images were encoded in 30 directions for each b-value with FOV of 236 × 236 mm^2^ and a matrix of 128 × 128. The total acquisition time was 25–35 min for the structural and DTI images. All images were resampled to 1$\times$1$\times$1 mm^3^.

### Image post-processing and registration

Fractional anisotropy (FA) and mean diffusivity (MD) parametric maps were calculated on a voxel-by-voxel basis, and the values of FA and MD were obtained as the average of all 30 directions of the highest b-value. Co-registration between the structural images and DTI parametric maps was performed based on geometric information using FSL (FLIRT-FMRIB’s Linear Image Registration Tool, Oxford, UK; http://www.fmrib.ox.ac.uk/fsl/) [39]. For every case, the T1WI, T2WI, FA and MD were co-registered with reference to FLAIR. Considering the variability of resolution in different images, the MRI images and masks were resampled into the same isotropic grid via linear interpolation. The intensity normalisation between cases was implemented in MATLAB R2014b (Mathworks, Natick, MA) using Z-score normalisation. Skull and scalp stripping was conducted via the BET brain extraction module in FSL, generating a better recognition for CNN mapping.

### Image segmentation, feature extraction and classification

**Fig. 1:**
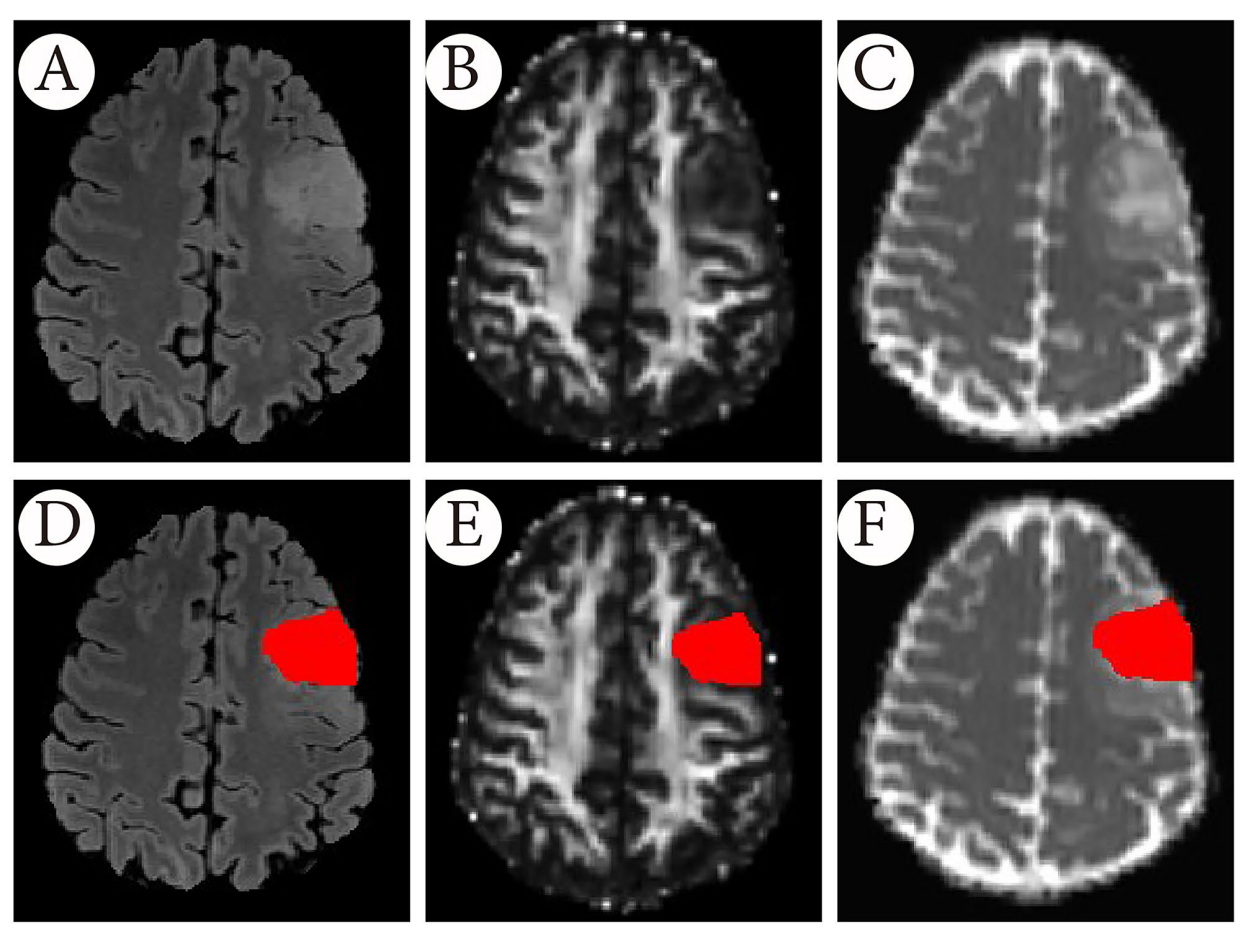
Graphical example of tumour segmentation process beginning from FLAIR and ending in co-registered FA and MD. Example slice of the ROIs drawn on the whole tumour on FA (B, E) and MD (C, F) parametric maps, based on parametric maps (D) which created from FLAIR (A)

Tumour regions of interest (ROIs) were outlined on 3D-FLAIR images using semiautomatic segmentation software (ITK-SNAP 3.6.0-rc1, Snake Interaction Mode) and verified by one radiologist with neuroimaging direction (J.Y, 10 years of reader experience) and one neuroimaging consultant (S.B.2, 10 years of consultant experience). Where considerable variability occurred in the outlined ROIs, final segmentation was based on consensus between the readers. The segmented volume contour was based on the hyperintense region in FLAIR images on multiple slices (Fig. 1), including any microscopic areas of cystic changes, calcification, hemorrhagic foci, and necrosis, but excluded large vessels within the tumour and macroscopic cysts and necrosis. To ensure the robustness and reliability of the data quality, another neuroimaging consultant (S.B.1, 12 years of experience) conducted a visual quality assurance (QA) check on the 30 randomly selected cases. Referent masks were derived for evaluating the overlap metrics. The Dice Similarity Coefficient (DSC) [40] was computed for each pair of masks, and acceptable data quality required the minimum DCS value > 0.95. The mean value of FA and MD in the contralateral normal-appearing white matter (NAWM) at centrum semiovale was also extracted using a standardised ROI for testing any collinearity of the DTI values with the age and gender of the patients. The tumour ROIs created from the anatomical images were subsequently applied to the DTI images.

**Fig. 2:**
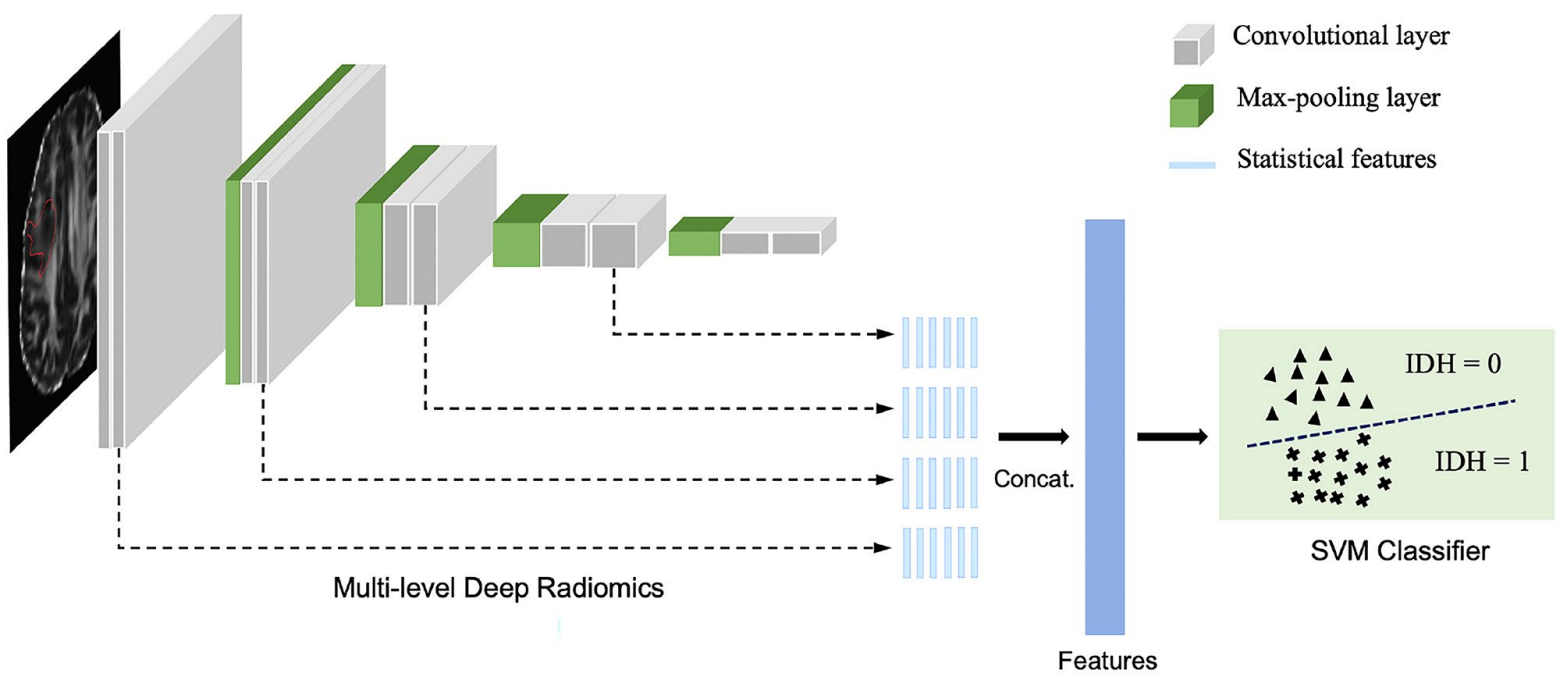
The feature extraction process was based on a pre-trained VGG [41] network and mutation status prediction was executed using an SVM [42] classifier. Six statistical features were extracted from each convolutional layer in a multi-level manner and were then concatenated into a feature vector

The feature extraction process was conducted in MATLAB R2021a. The experiments were carried out on Ubuntu 20.04 with an NVIDIA GeForce RTX 2080 Ti GPU and an Intel Core i7-9700 K 3.60 GHz CPU. For each subject with one MR modality, the tumour MR image was fed into the pre-trained VGG16 [41] model (see Fig. 2), and its radiomics features were extracted from multiple convolutional layers which learnt different levels of texture and shape information. From each feature map in the convolutional layer, six statistical features including mean, median, standard deviation, 5th and 95th percentiles and kurtosis, were extracted. Then all the features from multiple levels were concatenated into one feature vector. As the size of the feature vector grows considerably when the number of used layers increases, in practice, we extracted the radiomics from the first four convolutional layers. For example, when using the combination of FA and MD modalities, the feature vector size using the first three convolutional layers is 2 * 6 * (1 + 64 + 64 + 128) = 3084. The time required to extract all features from one case using the pretrained VGG16 model was estimated via CPU, and mean value over several test cases is 41.92 s (confidence intervals 41.30–42.70).

**Fig. 3:**
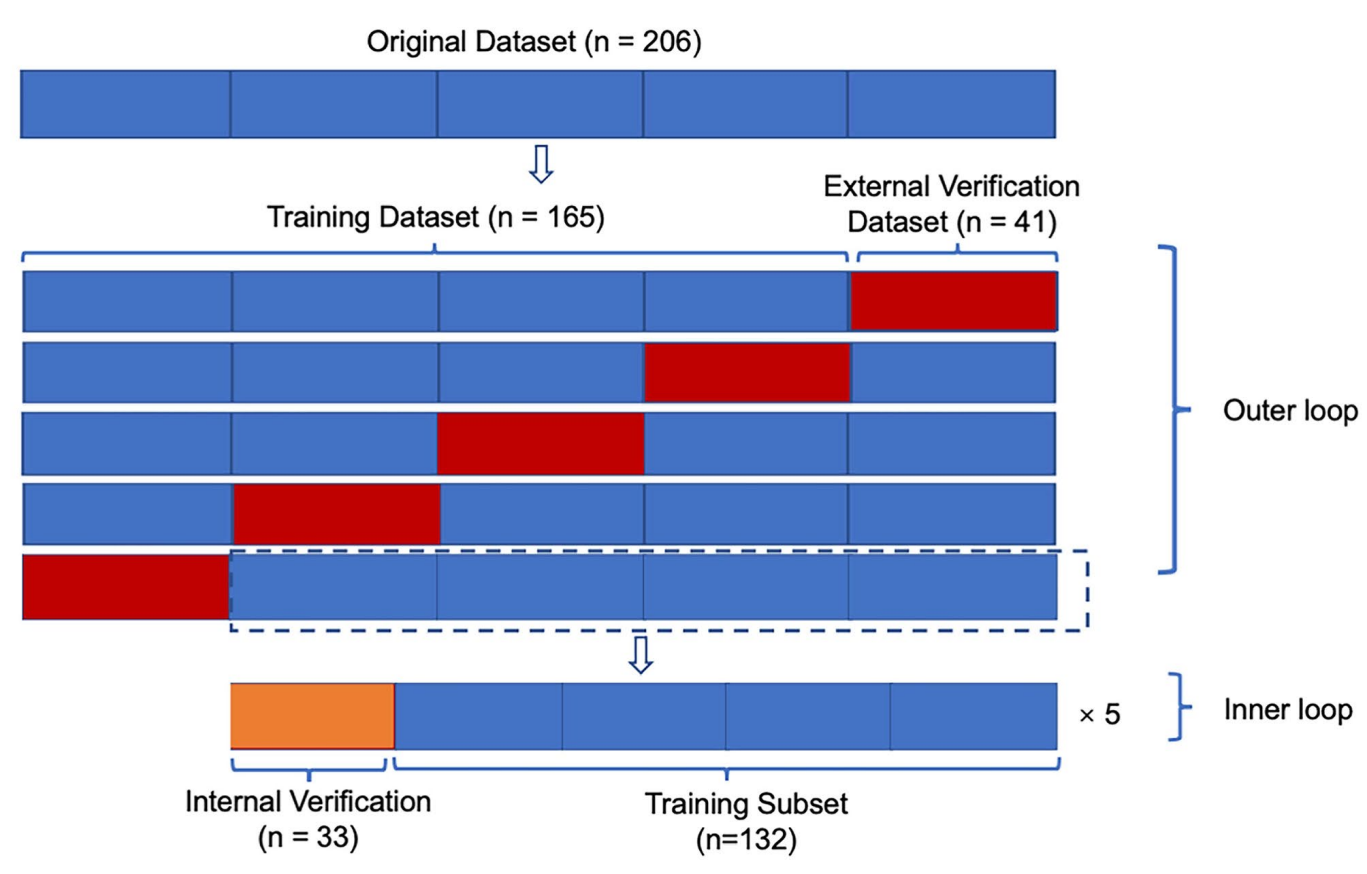
5-fold nested cross-validation. The kernel parameter is radial basis function (RBF), and penalty parameter C and γ values are determined by grid search in the internal loop of the nested cross validation stage, which were not fixed

In the classification stage, the features and their corresponding IDH mutation status were used to train an SVM [42], which is a top-performing classifier that constructed a decision boundary to separate the two groups based on the mutation status. The code for classification was implemented with MATLAB R2020b. The experiments were carried out on Ubuntu 18.04 with an NVIDIA GeForce RTX 2080 Ti GPU and an Intel Core i7-9700 K 3.60 GHz CPU. The kernel parameter is radial basis function (RBF). The outputs of SVM (the distance to the decision boundary) were then calibrated into posterior probabilities by training an additional sigmoid function [43]. A nested five-fold cross validation setting was used as the evaluation protocol with the inner loop for parameter selection and the outer loop for model evaluation (see Fig. 3), considering the nested cross-validation as an almost unbiased estimate of the true error and a suitable method for a small dataset [44]. In each fold of the nested cross-validation, the optimal hyper-parameters of the classifier were chosen based on an internal validation which is 20% of the training set. Specifically, we consider the recall, and F1-score as the evaluation metrics to monitor the classifier performance. The optimal parameters and kernel choice of SVM (C, γ) were found by grid search using the inner cross-validation and the outer one was used to assess the final prediction performance on the validation set in each fold.

### Statistical methods and evaluation metrics

Statistical analysis was performed using Python software (version 3.6). Given the wide age range of the patients and the reported age-related changes in FA and MD in white matter [45], we first checked whether there were any significant correlations between NAWM and patient age or gender using linear regression tests and Pearson’s chi square tests, respectively. A Welch two-sample t-test or a Mann-Whitney U-test, and a Pearson’s chi-squared test with Yates’ continuity correction were used to determine whether any of the clinical characteristics, including age and gender, were correlated to IDH mutation. Secondly, we assessed the predictive power of DTI, structural images and their different combinations by calculating the sensitivity and specificity of the algorithm for standalone and combined modalities. We also reported the receiver operating characteristic (ROC) curve and the area under the curve (AUC). In all cases, statistical significance was indicated at a level < 0.05.

## Results

### Clinical characteristics

**Fig. 4:**
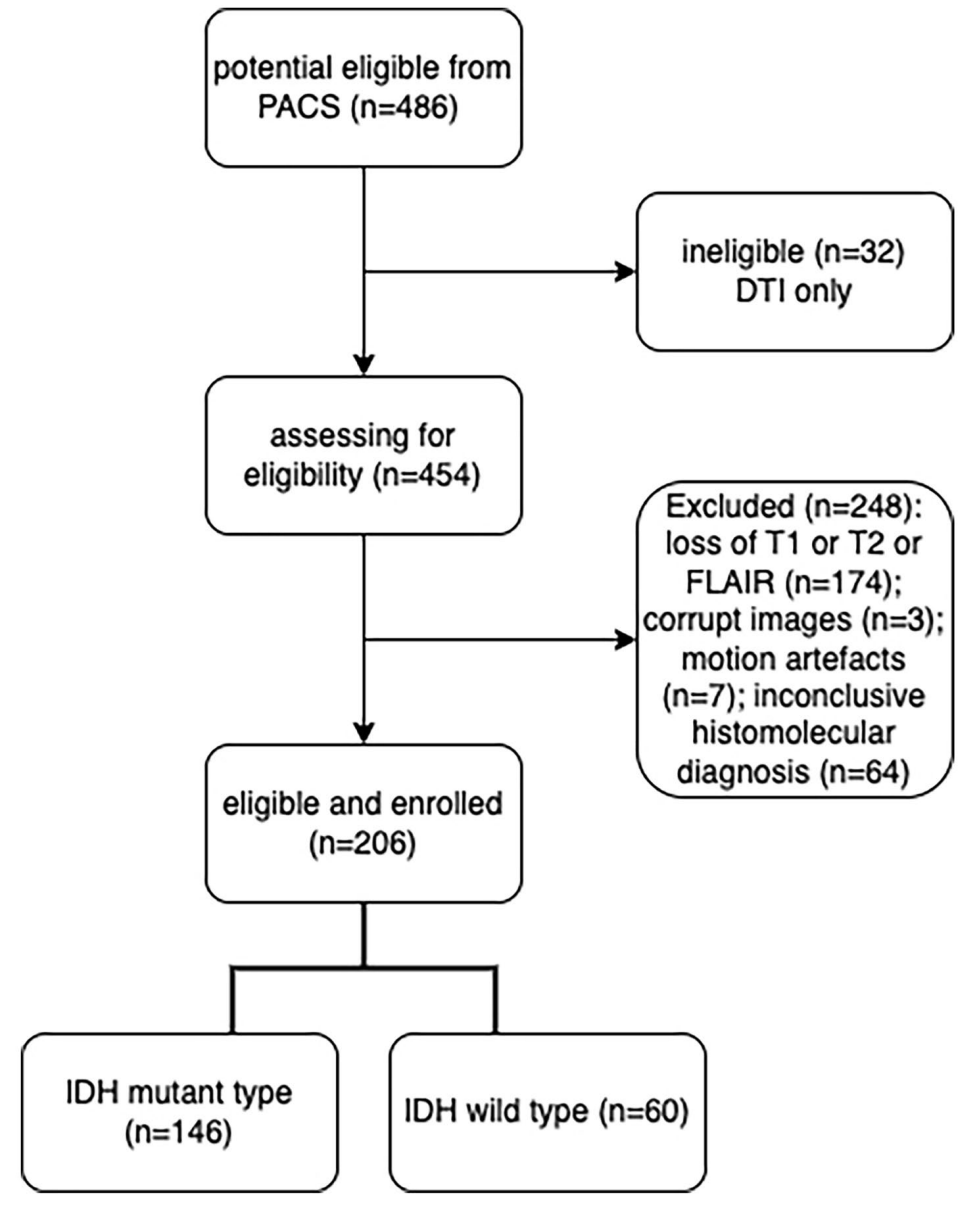
Participant flow

A cohort of 206 cases was eligible and enrolled for the IDH phenotypes analysis. The participant flow chart is shown in Fig. 4. The final cohort included 100 females and 106 males; 146 of the patients had IDH mutant and 60 had IDH wild-type gliomas. The clinical characteristics of our participants are summarised in Table 1. The mean age of all participating patients was 44.0 years (range: 19–77 years). There was no significant correlation between patients’ age and the FA and MD values of the NAWM (*P* = 0.06 and *P* = 0.66, respectively). Also, there was no significant correlation between patients’ gender and the FA and MD values of the NAWM (*P* = 0.44 and *P* = 0.59, respectively). Patients with IDH mutant gliomas (mean age$\pm \text{S}\text{D}:$ 39.1$\pm 8.7$ years) were younger than those with IDH wild-type gliomas (mean age$\pm \text{S}\text{D}:$ 55.1 $\pm 13.5$ years, *p* < 0.001). Gender was not correlated to IDH mutation type in our study.

**Table 1:** Clinical characteristics

	Total	IDH mutant	IDH wild type	*P* value
Number of cases	206	146	60	
Age (mean ± std, years)	44.0±12.8	39.1±8.7	55.1±13.5	0.00 §
Gender				0.20*
Female	100 (49%)	75 (51%)	25 (42%)	
Male	106 (51%)	71 (49%)	35 (58%)	

Note. §The difference between the two groups was evaluated using the Mann-Whitney U-test because the data was not normal distribution according to Shapiro-Wilk test. *The correlation between the two groups was evaluated using the Pearson’s Chi-squared test with Yates’ continuity correction

### Image quality assurance, comparison of DTI, structural MRI and their combinations

The DSC values in the checked masks were within acceptable limits, with a minimum DSC value of 0.98, the data was thus considered acceptable for further analysis. We first compared the predictive power of deep radiomics extracted from DTI (FA and MD), structural MRI (FLAIR, T1 and T2) and their combinations in automated classification of IDH mutation status. A fair comparison was achieved by reporting the performance on the same validation sets in nested five-fold cross validation in which the machine learning model was trained on the same patients. For each patient, deep radiomics features were extracted individually for each modality and their combinations, and then used to train a statistical SVM classifier.

As shown in Table 2, DTI itself brought highly discriminative information and achieved an AUC of 0.832 and sensitivity of 90.4%, while structural modalities attained an AUC of 0.730 and sensitivity of 92.5%. Combining DTI and structural modalities demonstrating an improvement in diagnostic performance with a higher AUC than the standalone techniques (0.846 vs. 0.832 and 0.730).

**Table 2:** Different combinations of imaging modalities revealed that DTI metrics (FA and MD) serve as effective biomarkers, and can be further enhanced by integrating structural modalities

Modality	True Positive	False Positive	True Negative	False Negative	Sensitivity	Specificity	AUC
FA + MD	132	27	33	14	0.904	0.550	0.832
T1 + T2 + FLAIR	135	28	32	11	**0.925**	0.367	0.730
FA + MD + FLAIR	133	25	35	13	0.911	0.583	0.846
FA + MD + T1 + FLAIR	133	26	34	13	0.911	0.567	0.835
FA + MD + T1 + T2 + FLAIR	135	26	34	11	**0.925**	0.567	0.846
FA + MD + T1 + T2 + FLAIR + age	133	23	37	13	0.911	**0.617**	**0.847**

In rows 2 and 5 in Table 2, it is evident that combining FLAIR images with DTI, could improve the AUC from 0.730 to 0.846 and specificity from 0.367 to 0.567, while maintaining the same sensitivity of 0.925.

**Fig. 5:**
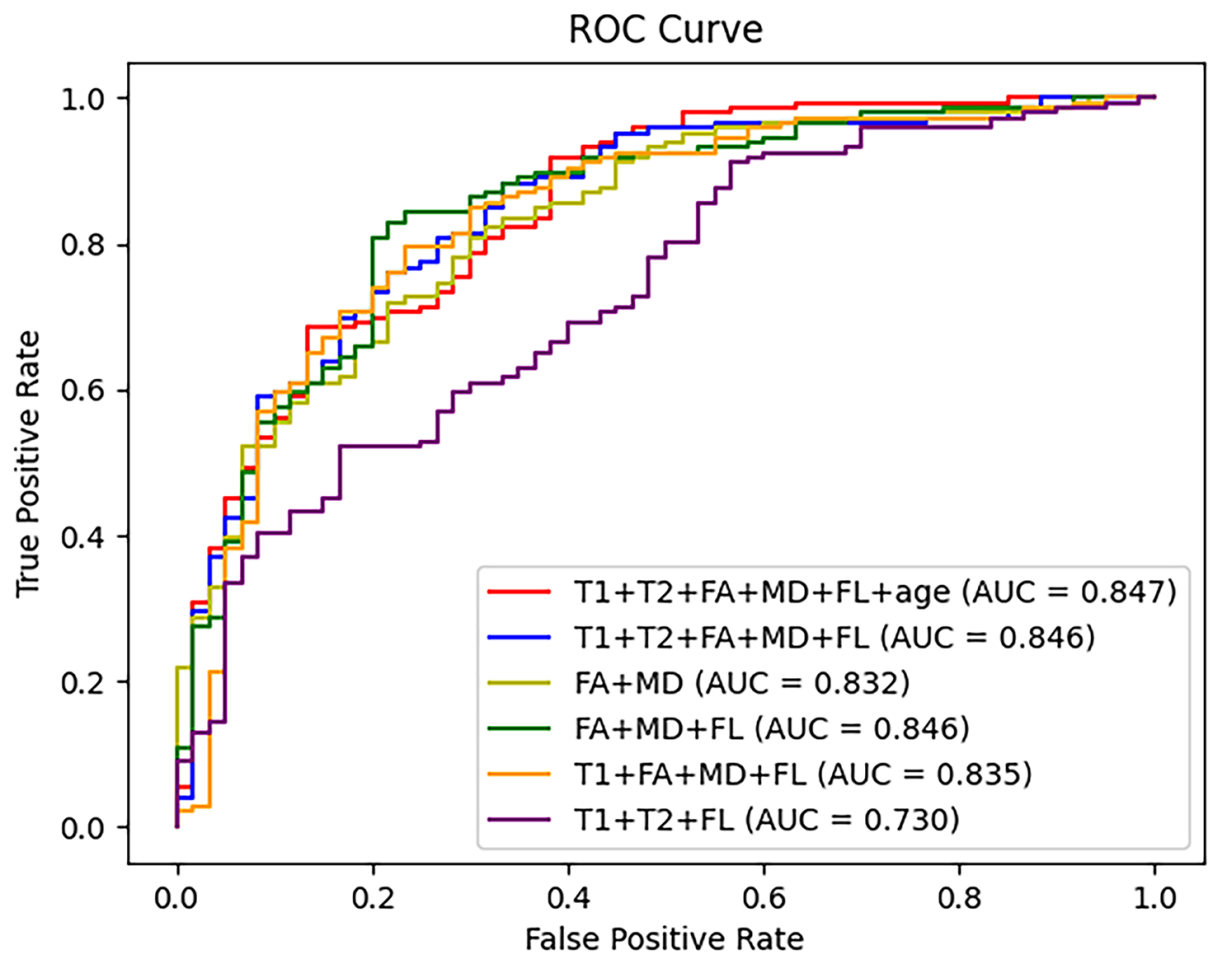
ROC curves of the performance using different combinations of imaging modalities. We observed that the information extracted from DTI and structural MR modalities were complementary in IDH mutation status prediction. Meanwhile, adding the variable of ‘age’ had significant contribution to the predictive performance

We further investigated the potential advantages of integrating the demographic information into the machine learning to enhance the predictive power. The combination of DTI with structural and patients’ age led to the highest AUC of 0.847 (see Fig. 5), along with the highest specificity of 61.7%. The diagnostic performance using the combination of imaging and demographics was significantly different when compared with the combination of DTI with structural images (*P* = 0.003) (Table 3).

**Table 3:** Statistics analysis of various combinations of imaging modalities and demographic information. The *p*-values were adjusted after correcting the false discovery rate under multiple hypotheses

Comparison	FA + MD vs. T1 + T2+ FLAIR	FA + MD + T1 + T2+ FLAIR + age vs. FA + MD	FA + MD + T1 + T2+ FLAIR + age vs. T1 + T2 + FLAIR	FA + MD + T1 + T2+ FLAIR + age vs. FA + MD+ T1 + T2 + FLAIR
*p*-value	< 0.0001	< 0.0001	0.0005	0.003

### Analysis on individual layers

To ensure the discriminativeness of the selected layers, we analysed the individual features extracted from layer 1 to layer 5 (Table 4). We concluded that the first four layers yielded comparable performance whilst the 5th layer showed a significant drop. Thus, only the first four layers were subsequently included.

**Table 4:** Analyses on the discriminativeness of involved features from FA and MD modalities in different layers. Layer 0 denotes traditional radiomics features [46] from input images

Layers number	0	1	2	3	4	5
AUC	0.813	0.832	0.825	0.833	0.835	0.801
Sensitivity	0.904	0.904	0.911	0.911	0.911	0.911
Specificity	0.533	0.550	0.533	0.550	0.567	0.364

### Feature visualisation

**Fig. 6:**
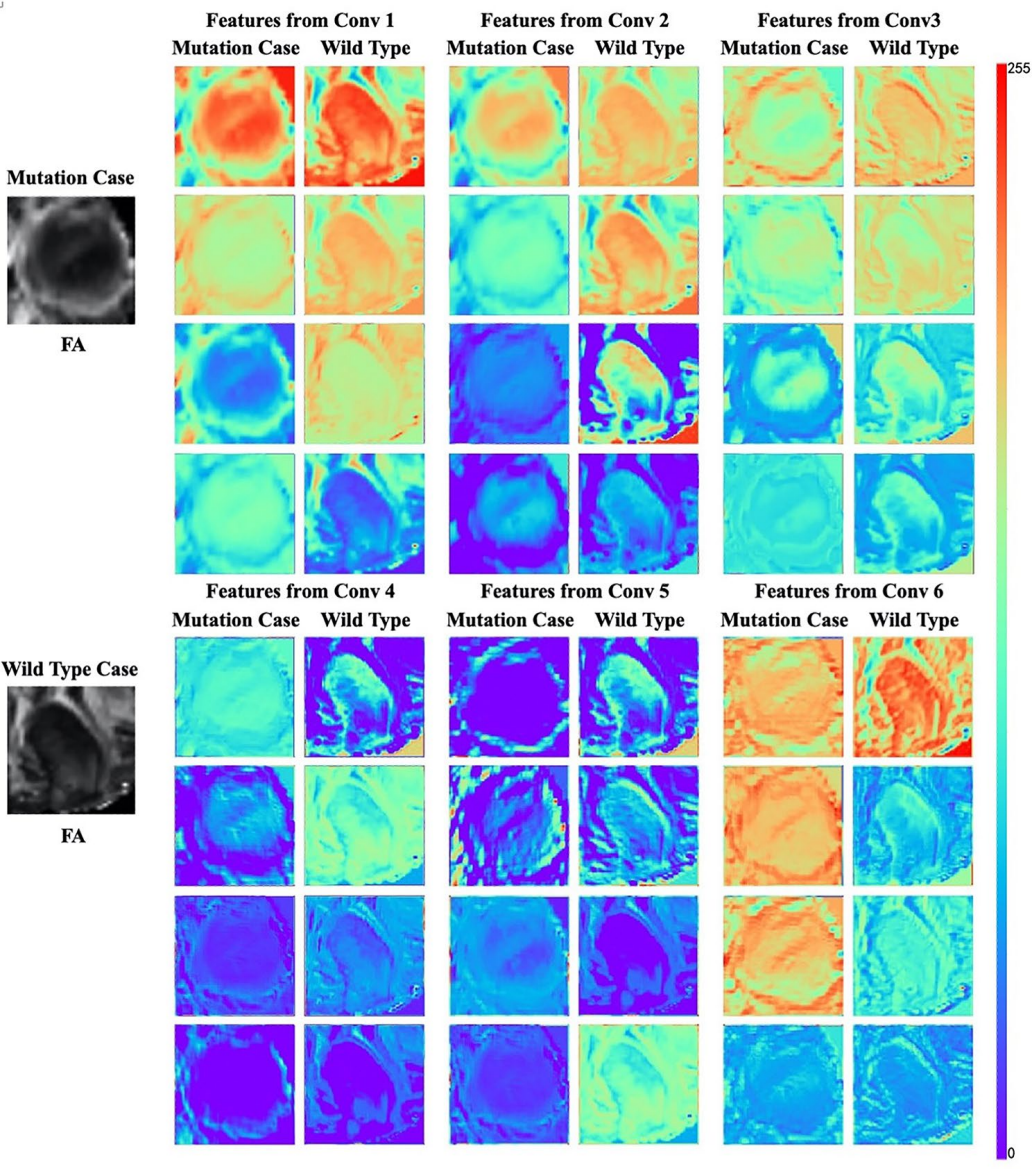
Visualisation of the features captured by each convolutional layer. We found that shape, edge, and texture information were captured in different layers

To assess the interpretability of the deep radiomics feature, we randomly selected four feature maps from each convolutional layer and visualised them in a heatmap. Figure 6 shows the results of one mutation case and one wildtype case. We found that the shape, boundary and texture of tumour were well visualised in different layers using the proposed computational approach. Apparently, such information may not be easily perceived and classified by humans reviewing the DTI images.

## Discussion

We present a deep radiomics approach to extract imaging-based features from DTI and structural images for the prediction of IDH mutation status of CNS WHO grade 2–4 gliomas within a single centre, multi-scanner setting. This effort builds upon CNN models, leveraging an SVM classifier with feature differentiablity, enabling both comparative and combinatory assessments of datasets. This differentiable model harnesses the potential of combining DTI and structural MRI for versatile, non-invasive IDH mutation status prediction in diverse clinical settings.

Our study demonstrates that DTI features are effective biomarkers independent from their structural MRI counterparts. Specifically, our preliminary results show that our predictive model achieved a higher diagnostic performance based on standalone DTI features compared to standalone structural features (sensitivity of 90.4%, specificity of 55.0% and AUC of 0.832 compared to 92.5%, 36.7% and 0.730 respectively). The combination of DTI and structural features yielded further improvement in diagnostic performance with a higher AUC (AUC = 0.846) compared to standalone DTI (AUC = 0.832) or structural (AUC = 0.730) features. Further enhancement in predictive power was observed following the incorporation of demographic information, namely patients’ age, with the combined DTI and structural imaging features, leading to the highest AUC (0.847). Our methodology is characterised by strengths and weaknesses, however, builds on previous studies and warrants further work in the field.

Our results indicate a strong correlation between DTI-derived imaging features based on FA and MD and IDH mutation status in gliomas. This is consistent with previous studies which highlighted the promising role of DTI in probing tumoural microstructure and predicting IDH mutation status in gliomas [10, 47–49]. We postulate that decreased MD values identified in IDH wild-type gliomas reflect increased cellularity in these tumours, which is a downstream effect of upregulation of the HIF1A pathway leading to increased hypoxia, higher angiogenesis, and cell proliferation [30, 50]. Another possible explanation for our results is that the MD and FA values may be influenced by the composition of the extracellular matrix. Evidence suggests that the increased levels of glycosaminoglycan hyaluronan found in astrocytomas and oligodendroglial tumours positively correlate with MD values [31]. Previous studies revealed that the tumour FA values were positively correlated with the proliferative potential of gliomas [51–53], and could be employed to distinguish IDH-mutant from wildtype gliomas [54, 55]. Hence, we believe that FA and MD may be sensitive to early pathological changes and tumour heterogeneity and can be reliable IDH status predictors, superior to conventional structural images.

Notably, previous studies inevitably used a nomenclature according to the WHO 2016 classification [29, 30, 54, 55], and some tumour types (previously known as “entities”) have been renamed but correspond to the same molecular profile. For example, astrocytoma IDH-mutant CNS WHO grade 2, 3, and 4 tumours correspond to the previous diffuse astrocytoma IDH mutant WHO grade II, anaplastic astrocytoma IDH-mutant WHO grade III, and glioblastoma IDH-mutant WHO grade IV, respectively. Likewise, oligodendroglioma IDH-mutant and 1p/19q codeleted CNS WHO grade 2 and 3 tumours, correspond to oligodendroglioma IDH-mutant and 1p/19q codeleted WHO grade II, or anaplastic oligodendroglioma IDH-mutant and 1p/19q codeleted WHO grade III, respectively. Importantly, at our centre, we applied an integrated diagnosis for histologically low-grade appearing IDH-wildtype gliomas with TERT promoter mutation and other molecular features corresponding to glioblastoma IDH-wild-type WHO grade IV according to the 2016 classification and the cIMPACT-Now guidelines [35, 36]. Thus, in contrast to many previous studies, our study investigates differences in DTI metrics between IDH mutant and wildtype gliomas within a cohort which pragmatically incorporates all glioma grades (CNS WHO grade 2–4) [10, 47–49].

We aspired to provide a generalisable tool that can be applied for non-invasive classification of IDH-mutation status in different technical parameters. To accommodate this, we included data derived from three 3T MR scanners. In contrast, a large portion of previous work was conducted in a single scanner design [28, 29, 55]. However, a well-trained model from a single scanner may not be applicable to a different scanner or centre, hence impeding its clinical utilisation. For example, Li et al. reported successful application of radiomics for predicting the IDH mutation status in LGGs, achieving a remarkable AUC of 95% [17]. Nevertheless, such results may be rather optimistic due to a confined well controlled cohort, with limited applicability in different scanner and population settings. Our study utilized data from three different scanners and was conducted at a single institution, specifically a national tertiary neuro-oncology reference centre with strengths including comprehensive care and a remarkable ethnic diversity of the patient population. Our study was intended to be further generalisable through image normalisation and fine-tuning, though this may be the reason for the lower AUC. Additionally, The observed discrepancy in specificity (56.7%) despite commendable AUC (0.846) and sensitivity (92.5%) could be attributed to several factors: Predicting IDH mutation status may be a complex task with overlapping features between mutation and non-mutation cases. The model might identify certain imaging features associated with the mutation but struggle with specific characteristics that distinguish non-mutation cases; the features used for prediction may have a higher impact on sensitivity than specificity. The model may be more sensitive to characteristics associated with IDH mutation, leading to higher true positive rates at the expense of specificity. In the context of gliomas harboring IDH mutations, there might be a clinical expectation to identify such cases for tailored therapeutic interventions. This could include targeted therapies like IDH inhibitors, DNA repair inhibitors, and immunotherapy, which may benefit relapsed or refractory IDH-mutant glioma patients, particularly to reduce the long-term toxicities, including cognitive decline caused by conventional treatments. Our model with noteworthy AUC and sensitivity may help in the above-mentioned situation. Finally, the acquired satisfactory and encouraging results are in accordance with other multi-scanner and multi-centre studies, which have reported realistic generalisable prediction rates for non-invasive IDH mutation status determination [19, 56, 57].

To make our technique more agnostic and less dependent on input for multiple modalities, we chose to base segmentations on the universally employed FLAIR images. Currently, other methodologies employ volumetric measurements based on enhancing tissue on post contrast T1WI. However, tumour cells have been described to exist outside the contour of the enhancing tumours being part of the complex tumour micro-environment, causing the notorious peritumoural infiltration zone in the gliomas [58]. FLAIR images may be more representative of the actual extent of tumour infiltration, albeit in combination with vasogenic oedema in the peritumoural brain parenchymal zone (PBZ) [59]. In our study, IDH wild-type gliomas did not demonstrate significantly larger volumes in our ROI compared with their IDH mutant counterparts. This may be partly attributed to more pronounced oedema characterising IDH wild-type neoplasms and in contrast larger solid tumour components characterising IDH mutant type gliomas, consistent with previous studies [60, 61]. Interestingly, standalone FLAIR sequences proved not to be beneficial for the prediction of IDH phenotype, even when combined with DTI. A possible explanation is that T2WI and pre/post contrast T1WI sequences provide complementary information regarding the tumour microvascular proliferation and/or necrosis, which are established diagnostic and prognostic biomarkers for IDH wildtype gliomas [11, 62, 63]. Such an explanation is supported by our best performing model which is multimodal and incorporates the combination of FA, MD, T1WI, T2WI and FLAIR.

Our study encompasses distinct methodological differences to previous studies and contributes additional knowledge to the field [47–49]. In most studies, the size of the DTI dataset used in identifying IDH mutation status is relatively small compared to the publicly available structural images (such as FLAIR, T1 and T2) [57, 64], because DTI is not routinely applied in general clinical practice. To get robust estimation of the proposed method, we adopted nested five-fold cross validation to alleviate the potential negative effect of insufficient number of samples. We included a small fraction of data that were randomly split from the original dataset and kept untouched for use as a test dataset in our 5- fold nested cross-validation (see Fig. 3) [64–66]. Prior to our proposal for a deep radiomics approach, we attempted several end-to-end deep learning techniques, such as 2D and 3D DenseNet, which however did not yield promising outcomes in our multi-scanner cohort (data is not provided). This likely reflects limitations of training a deep neural network on heterogeneous datasets with limited data samples. We followingly sought a hybrid approach by combining deep learning with traditional machine learning. We claim that such a combination benefits feature extraction given the small number of samples. Model performance often degrades using multi-scanner and multi-centre cohorts, as shown by similar work using the Cancer Imaging Archive (TCIA) which studied structural images rather than DTI [56, 57]. On the other hand, we have an unbalanced cohort as expected because IDH1 or IDH2 mutations are common in 70–80% of gliomas [10, 29]. Admittedly, the unbalanced cohort can negatively affect the classifier. To account for this, we used ‘class weights’ for weighting strategy considering class imbalance in the SVM. Specifically, smaller weight (= 1) is assigned to the majority class (IDH mutant) and larger weight (= 3) to the minority class (IDH-wildtype). Before weighting, our DTI + structural model had sensitivity = 0.911, specificity = 0.526, AUC = 0.817 and after weighting these slightly improved (sensitivity = 0.925, specificity = 0.567, AUC = 0.846). In addition, to enhance the validation of our approach, we tested our final models from five folds on a balanced test set comprising 20 patients added subsequently to the study. This test set was equally divided to represent each mutation status, with 10 patients per category. The predictions on the separate test were obtained by an ensemble of the average of five models form five folds. The sensitivity, specificity, precision, AUC are 1.000, 0.602, 0.601, and 0.834 respectively, demonstrating the robustness of our method.

There are a few limitations in our work. First, we extracted a high number (> 3000) of feature variables from the imaging data and this may impede the inclusion of other variables, such as demographical information, which could potentially enhance the predictive power. Dimensionality reduction techniques such as principal component analysis [67] shall be investigated in future work so that the role of clinical variables could be enhanced. Autoencoder [68] or variational autoencoder [69] might be a good alternative for low-dimensional feature exaction in our future work. Second, although we performed analysis based on a multi-scanner cohort, the effect of the scanner to the performance of the machine learning model and the generalisability of the method to data from unseen scanners, have not been formally investigated. To account for potential differences in FA and MD values between different scanners, we performed a comparative leave-one-scanner-out calculation. We removed the 11 cases conducted by Skyra 3T MR scanners (b value = 1400 s/mm2) from the training dataset in our DTI and structural images model, with resulting sensitivity = 0.870, specificity = 0.553 and AUC = 0.765. *P* = 0.00. Our comparative estimation demonstrates that different b values of DTI scan protocol may affect the outcomes of our prediction model. Further study should be performed regarding the impact of different b values to the generation of DTI radiomic model. Third, the CDKN2A/B mutation was not assessed in all IDH-mutant samples in our cohort; since the presence of CDKN2A/B homozygous deletion results in CNS WHO grade 4 rather than grade 2/3 tumour staging, even in the absence of microvascular proliferation or necrosis [62]. However, as there is a significant overlap of histological high-grade features (i.e. necrosis and microvascular proliferation) with the molecular feature of CDKN2A/B homozygous deletion, the risk of misclassification is minimal in our cohort. For IDH-wildtype gliomas, whether the CDKN2A/B status is diagnostically and prognostically irrelevant has not been systematically examined. In the meantime, all IDH mutant tumours diagnosed at our institution are being examined with Illumina epic arrays which provide readout also for the copy number profile, i.e. chromosomal gains, losses, and gene amplifications and deletions such as 1p/19q codeletion in oligodendroglioma, or for IDH mutant astrocytomas, the CDKN2A/B, homozygous deletion to establish a CNS WHO grade 4 in IDH-mutant astrocytomas. Fourth, recent deep learning methods, such as Transformer [70], which can capture long-range dependencies, might learn effective feature representation in the 3D multi-modality setting. Hematological factors (e.g., CD4 + T cells, neutrophils, the neutrophil/lymphocyte ratio [71, 72]) and other MRI methods (e.g., dynamic susceptibility contrast magnetic resonance imaging, perfusion, diffusion kurtosis images [34, 73, 74]), have prognostic value for glioma. Therefore, performing a reader analysis to compare the diagnostic performance of our CNN-SVM model would be meaningful. Additionally, refining the deep learning method and incorporating more biomarkers and MRI parameters are inspiring aims for future work.

## Conclusion

This study demonstrates that the integration of structural MRI and DTI features improves prediction performance compared with structural MRI or DTI alone, by using a deep radiomics approach. Further enhancement of diagnostic accuracy can be achieved by incorporating demographic information, specifically patients’ age, in the model. The applied CNN-SVM network showed potential for an automated prediction of IDH mutation status, and our work proposes a clinically feasible approach for optimisation of patient management through non-invasive IDH status prediction in gliomas. Nevertheless, the challenge of improving diagnostic accuracy remains and further research is required to validate and increase the model performance in a multi-centre background.

## Data Availability

The dataset used and analysed in current study, and the code for machine learning and the radiomics analysis can be acquired from the corresponding author upon reasonable request.

## Abbreviations

IDH: isocitrate dehydrogenase
DTI: diffusion tensor imaging
CNNs: convolutional neural networks
SVM: support vector machine
CNS WHO: Central Nervous System World Health Organization
FA: fractional anisotropy
MD: mean diffusivity
FLAIR: fluid attenuated inversion recovery
TERT: telomerase reverse transcriptase
EGFR: epidermal growth factor receptor
DNA: deoxyribonucleic acid
ATRX: alpha-thalassemia/mental retardation X-linked
AUC: area under the curve
LGGs: low-grade gliomas
MGMT: O^6^-methylguanine-DNA methyltransferase
qPCR: quantitative polymerase chain reaction
MRS: magnetic resonance spectroscopy
MR: magnetic resonance
MRI: magnetic resonance imaging
3D: three-dimensional
T1WI: T1-weighted images
T2WI: T2-weighted images
ADC: apparent diffusion coefficient
DKI: diffusion kurtosis imaging
TR: repetition time
TE: echo time
FOV: field of view
SS: single-shot
SE: spin-echo
EPI: echo-planar imaging
ROIs: regions of interest
QA: quality assurance
DSC: Dice Similarity Coefficient
NAWM: normal-appearing white matter
ROC: receiver operating characteristic
TCIA: the Cancer Imaging Archive
